# Augmenting Internet-Based Cognitive Behavioral Therapy for Major Depressive Disorder With Transcranial Infrared Laser Stimulation

**DOI:** 10.1016/j.bpsgos.2025.100449

**Published:** 2025-01-09

**Authors:** Douglas W. Barrett, Christopher G. Beevers, F. Gonzalez-Lima

**Affiliations:** Departments of Psychology, Psychiatry and Behavioral Sciences, and Institute for Neuroscience, The University of Texas at Austin, Austin, Texas

**Keywords:** Cognitive behavioral therapy, Cognitive enhancement, Deprexis, Major depressive disorder, Prefrontal cortex stimulation, Transcranial infrared laser stimulation

## Abstract

**Background:**

Transcranial infrared laser stimulation (TILS) is a noninvasive form of photobiomodulation that facilitates prefrontal energy metabolism and oxygenation, resulting in cognitive-enhancing effects. Cognitive behavioral therapy is a mainstream treatment for major depressive disorder. This is the first study to investigate whether TILS would augment the antidepressant effects of internet-based cognitive behavioral therapy.

**Methods:**

Sixty participants with major depressive disorder were given access to Deprexis, a form of internet-based cognitive behavioral therapy, for 12 weeks. After the first 2 weeks, the 40 participants who had improved at least 10% in depressive symptoms from baseline as measured by the Quick Inventory of Depressive Symptomatology–Self-Report were randomly assigned to Deprexis in combination with TILS or sham/placebo. There were no significant group differences in demographics or initial depression data.

**Results:**

There was a 43% reduction in Quick Inventory of Depressive Symptomatology–Self-Report scores in the sham group from the initial score to the week 12 score, while adding TILS as an adjunct therapy resulted in a reduction of 56%. Therefore, TILS resulted in an additional 30% reduction in Quick Inventory of Depressive Symptomatology–Self-Report scores ([56−43]/43 = 30%). Participants who received TILS to the right forehead once a week for 4 weeks showed a significantly greater reduction of depressive symptoms than participants who received sham/placebo. Participants reported no adverse effects.

**Conclusions:**

While Deprexis alone significantly reduced depression scores in the placebo control group, this beneficial effect was augmented with the addition of TILS as an adjunct therapy. Additional research that pairs neuroenhancement methods such as TILS with cognitive interventions may reveal the potential to improve treatment outcomes in depression and other psychiatric disorders.

## Plain Language Summary

Transcranial infrared laser stimulation (TILS) is a safe and noninvasive form of photobiomodulation that can be used to increase brain activity in the frontal lobes, resulting in enhanced cognition and emotional regulation. Deprexis is a form of internet-based cognitive behavioral therapy for treating depression. TILS administration to the forehead had a synergistic effect on improving depression symptoms when combined with Deprexis.

Cognitive behavioral therapy (CBT) emphasizes correcting negatively biased thinking, increasing behavioral activation, and reengaging with enjoyed activities. CBT is effective in treating major depressive disorder (MDD) (1). Deprexis, which is one form of internet-based CBT, may be a useful MDD treatment (2, 3, 4, 5, 6, 7) that is more effective when used in combination with other treatments (5).

Transcranial infrared laser stimulation (TILS) is a form of photobiomodulation that involves noninvasive administration of near-infrared light to the forehead [reviewed in Gonzalez-Lima (8)]. TILS has been shown to facilitate prefrontal energy metabolism and oxygenation (9,10), resulting in multiple cognition-enhancing effects (10, 11, 12, 13). We have shown that TILS enhances the effects of attention bias modification (ABM) (14), which is another cognitive intervention for depression. TILS applied to the right forehead, but not the left, resulted in significant symptom improvement. This beneficial effect of TILS was limited to the upper third of participants who showed an antidepressant response to ABM of at least 10%. However, photobiomodulation as a standalone intervention has limited benefits in MDD (15,16). Taken together, these results suggest that TILS may have a neuroenhancing effect on cognition and emotion that is synergistic with other interventions and may therefore be used to improve the effectiveness of MDD treatments such as CBT.

The purpose of this study was to determine whether TILS would augment the antidepressant effects of Deprexis for participants with MDD. We administered active and placebo TILS to the right forehead of participants who showed an improvement of at least 10% in depressive symptoms from baseline after 2 weeks of Deprexis treatment. We hypothesized that participants who received active TILS would show a greater reduction of depressive symptoms than participants who received placebo TILS.

## Methods and Materials

All procedures were approved by the University of Texas at Austin Institutional Review Board (IRB No. 2015-09-0100) and complied with the National Institute of Health and Declaration of Helsinki guidelines on human research.

### Participants

Participants were 60 adults from the Austin community who had a current MDD diagnosis determined by eligibility screening. Inclusion criteria included the following: 1) 18 to 45 years old; 2) ability to speak, read, and understand English fluently; 3) own a smartphone and able to receive emails on their phone; and 4) current depressive episode as determined by scoring ≥16 on the Center for Epidemiological Studies-Depression screening (17). Exclusion criteria included the following: 1) serious medical complications, including conditions that change electrical functioning in the brain (e.g., cancer, diabetes, epilepsy, head trauma, history of brain surgery, neurocognitive impairment, stroke, transient ischemic attack); 2) comorbid psychiatric disorders, excluding anxiety disorders; and 3) reporting of suicidal intent within the last 6 months.

Eligible participants came to the University of Texas’s Mood Disorders Laboratory for an initial meeting. Personnel provided participants with detailed oral and written descriptions of all study procedures and obtained informed consent. Table 1 shows demographic and clinical depression data for the participants.

**Table 1:** Demographic and Clinical Characteristics of Study Participants

Demographic Data	All Participants	Sham Group	TILS Group	Comparison
Age, Years	23.5 (4.8)	23.7 (5.1)	23.2 (4.4)	*U*_38_ = 197, *p* = .96^a^
Gender				
Female	34 (85%)	18 (85.7%)	16 (84.2%)	χ^2^_2_ = 1.22, *p* = .54^b^
Male	5 (12.5%)	3 (14.3%)	2 (10.5%)	
Nonbinary	1 (2.5%)	0 (0%)	1 (5.3%)	
Education, Years	14.4 (2.3)	14.0 (2.1)	14.8 (2.5)	*U*_38_ = 165, *p* = .33^a^
Race				
African American/Black	4	2	2	χ^2^_3_ = 5.25, *p* = .16^b^
Asian	5	5	0	
Multiracial	4	2	2	
White	27	12	15	
CES-D	40.3 (6.5)	41.2 (7.1)	39.3 (5.8)	*U*_38_ = 174, *p* = .50^a^

Values are presented as mean (SD), *n* (%), or *n*. Age and education are in years (through high school = 12 years, undergraduate = 16 years, masters = 18 years, doctorate = 20 years). CES-D initial score was used as one of the inclusion criteria.

CES-D, Center for Epidemiological Studies-Depression; TILS, transcranial infrared laser stimulation.

a Mann-Whitney *U* test (2 tailed) for comparison of group means.

b χ^2^ test for comparison of frequencies.

### Experimental Design

Researchers introduced the Deprexis program, and participants were given access to Deprexis for 12 weeks. The Deprexis program is self-guided, so participants determine how often they access the material. Deprexis consists of 10 content modules representing different psychotherapeutic approaches plus 1 introductory and 1 summary module, each of which can be completed in 10 to 60 minutes, depending on the user’s reading speed, interest, motivation, and individual path through the program, as detailed in Meyer *et al.* (2). While participants engaged with the Deprexis program, they completed the Quick Inventory of Depressive Symptomatology–Self-Report (QIDS-SR) each week, which served as our primary outcome measure to determine treatment efficacy. The QIDS-SR is a 16-item self-report measure of depressive symptom severity that is highly reliable, internally consistent, and sensitive to symptom change (18).

Participants whose QIDS-SR scores improved by at least 10% from baseline during the first 2 weeks of Deprexis treatment were eligible to receive 4 weekly TILS sessions and were randomly assigned to either the TILS group (*n* = 19) or the sham/placebo group (*n* = 21). Participants whose QIDS-SR scores did not improve by 10% from baseline (*n* = 19) were not eligible for TILS but continued to have access to the Deprexis program.

TILS was administered in the Gonzalez-Lima laboratory at the University of Texas once a week for 4 weeks. None of the TILS sessions were administered proximal to Deprexis session usage. Participants received either TILS or sham/placebo for 8 minutes during each weekly session. TILS consisted of applying light of a specific wavelength (1064 nm) using a laser (HD Laser; Cell Gen Therapeutics LLC), which is Food and Drug Administration–cleared as safe for use in humans. Both participants and experimenters wore protective eyewear, although the administrators of the TILS were careful not to shine the light in or near the eyes.

The same TILS parameters (Table 2) as in our previous studies (10, 11, 12,14,19) were used: The diameter of the circular forehead surface receiving TILS was 4 cm; the irradiance (power density) was 250 mW/cm^2^; and the fluence (energy density) was 60 J/cm^2^. In each TILS session, participants received eight 1-minute treatments to the forehead aimed at the right anterior prefrontal cortex (PFC) (Fp2 in the 10-20 electroencephalography coordinate system).

**Table 2:** Transcranial Infrared Laser Stimulation Parameters

Parameter	Value
Wavelength	1064 nm
Diameter of Stimulation Area	4 cm
Irradiance—Power Density	250 mW/cm^2^
Fluence—Energy Density	60 J/cm^2^
Stimulation Time	8 minutes
Stimulation Site	Fp2 (right anterior prefrontal cortex)

**Figure 1:**
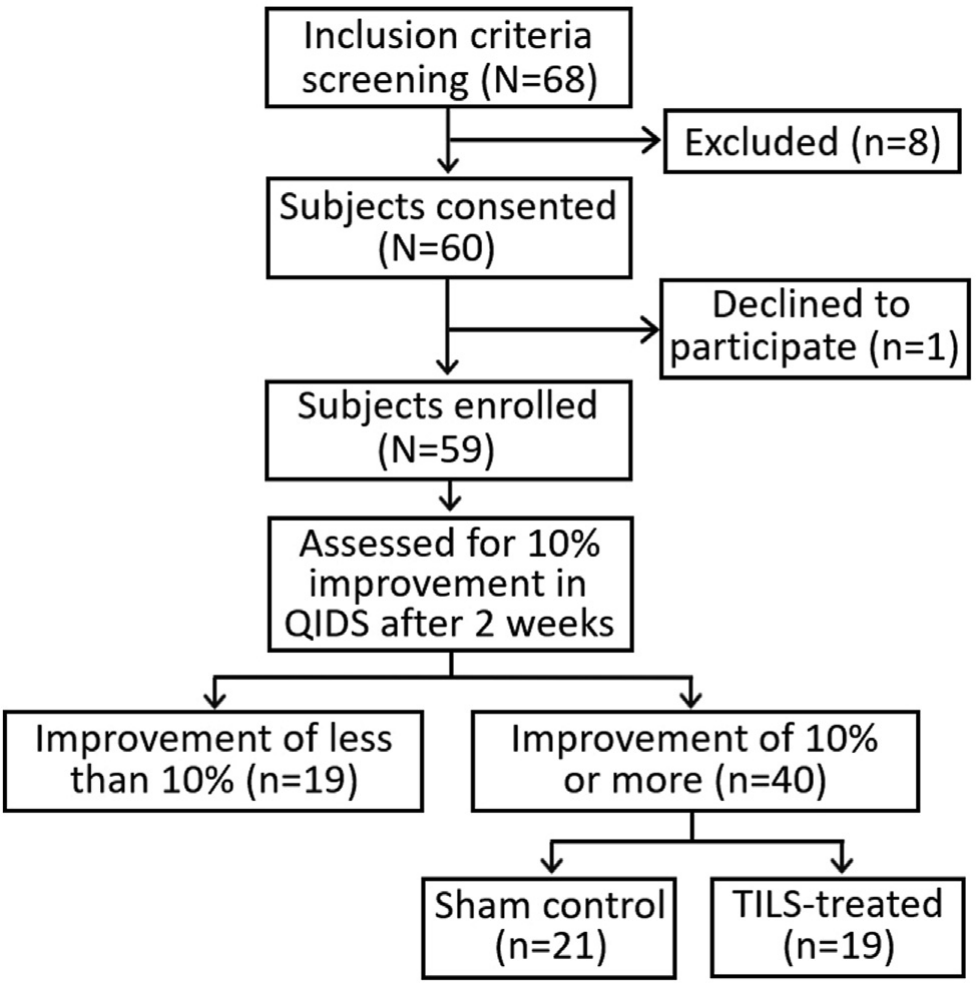
CONSORT (Consolidated Standards of Reporting Trials) diagram showing participant progress. QIDS, Quick Inventory of Depressive Symptomatology; TILS, transcranial infrared laser stimulation.

This TILS application has been estimated to penetrate into the gray matter of the human anterior prefrontal cortex (20) and upregulate mitochondrial energy metabolism (cytochrome c oxidase) and tissue oxygenation (oxygenated hemoglobin) (9,10). Participants with the sham/placebo control underwent the same procedure, i.e., received the same instructions and had the laser positioned in the same place at the forehead. However, the sham/placebo group received only a 5-second laser to the forehead followed by 55 seconds of laser off for each 1-minute cycle. This 5-second interval is sufficient to provide a sensation of slight heat (active sham/placebo) at the onset of each 1-minute cycle, using a fraction (1/12th) of the energy received by the experimental group, but it had no cognitive effects in our previous study (11), in which placebo-group participants were equally likely to guess that they were in the TILS versus placebo groups after the experiment. Following each session, participants were asked whether they experienced any perceived side effects (physical or psychological) from the TILS treatment. Figure 1 shows a CONSORT (Consolidated Reporting Standards of Reporting Trials) diagram with participant enrollment and random group assignment, and Table 3 shows the experimental timeline.

**Table 3:** Experimental Timeline

Week	Stage
0	Initial visit: screening, informed consent, Deprexis instruction, QIDS-SR
1	Deprexis, QIDS-SR
2	Deprexis, QIDS-SR, assessment for improvement, group assignment
3	Deprexis, TILS/placebo treatment, QIDS-SR
4	Deprexis, TILS/placebo treatment, QIDS-SR
5	Deprexis, TILS/placebo treatment, QIDS-SR
6	Deprexis, TILS/placebo treatment, QIDS-SR
7	Deprexis, QIDS-SR
8	Deprexis, QIDS-SR
9	Deprexis, QIDS-SR
10	Deprexis, QIDS-SR
11	Deprexis, QIDS-SR
12	Deprexis, QIDS-SR

QIDS-SR, Quick Inventory of Depressive Symptomatology–Self-Report; TILS, transcranial infrared laser stimulation.

### Statistical Analysis

Data from 40 participants who met the criterion of 10% improvement after 2 weeks of Deprexis were analyzed (19 TILS and 21 placebo). A power analysis (alpha = 0.05, power = 0.80) indicated that a group size of 15 to 20 participants was adequate to reveal significant effects of TILS on cognition and emotion (11,12). Demographic data and initial clinical depression data were compared between the TILS and sham groups using the Mann-Whitney *U* test for nonparametric data or χ^2^ tests for frequency data.

All QIDS-SR data were normally distributed. Missing data comprised <25% because we had a response rate of 76% overall for the 40 participants. Missing QIDS-SR values were handled using multiple standard statistical approaches. First, we used a last observed value carried forward method for a repeated-measures analysis of variance (ANOVA) including the 12 weeks of Deprexis treatment. We also used a second approach, mean imputation, in which missing values are replaced with the mean observed values for that participant. We used repeated-measures ANOVA and 2-tailed *t* tests to compare the group means for the 3 phases of the experiment: pre-TILS (week 0), during TILS (weeks 3–6), and post-TILS (weeks 7–12). Effect sizes were calculated as η_p_^2^ for the repeated-measures ANOVAs and Cohen’s *d* for *t* tests. All these approaches resulted in comparable findings in terms of statistical significance and effect sizes, which are reported separately in the Results.

## Results

### General Effects

There were no significant differences between groups for the demographic and initial clinical depression data (Table 1). Participants reported no adverse effects from TILS or sham.

The QIDS-SR data indicated that all participants improved over time. Within the placebo control group alone, a repeated-measures ANOVA (1 factor: weeks) over weeks 1 to 12 revealed a significant weekly reduction in QIDS-SR scores (*F*_11,220_ = 4.45, *p* < .001, η_p_^2^ = 0.182), indicating that Deprexis alone significantly reduced depression symptoms. There was a 43% reduction in QIDS-SR scores in the sham group from the initial score to week 12, while adding TILS as an adjunct therapy resulted in a reduction of 56%. Therefore, TILS resulted in an additional 30% reduction in QIDS-SR scores ([56−43]/43 = 30%). TILS versus sham group differences were statistically significant whether we analyzed the data using last observed value carried forward or mean imputation methods, as described below.

### Mean Effects of TILS Compared With Sham

TILS-treated participants showed significantly greater improvement in QIDS-SR scores than sham-treated participants using the last observed value carried forward method. A repeated-measures ANOVA (2 factors: weeks and group) on QIDS-SR scores during the 12 weeks of Deprexis treatment revealed a significant main effect of weeks (*F*_11,418_ = 10.3, *p* < .001, η_p_^2^ = 0.214) and a significant main effect of group (*F*_1,38_ = 4.98, *p* = .032, η_p_^2^ = 0.116).

**Figure 2:**
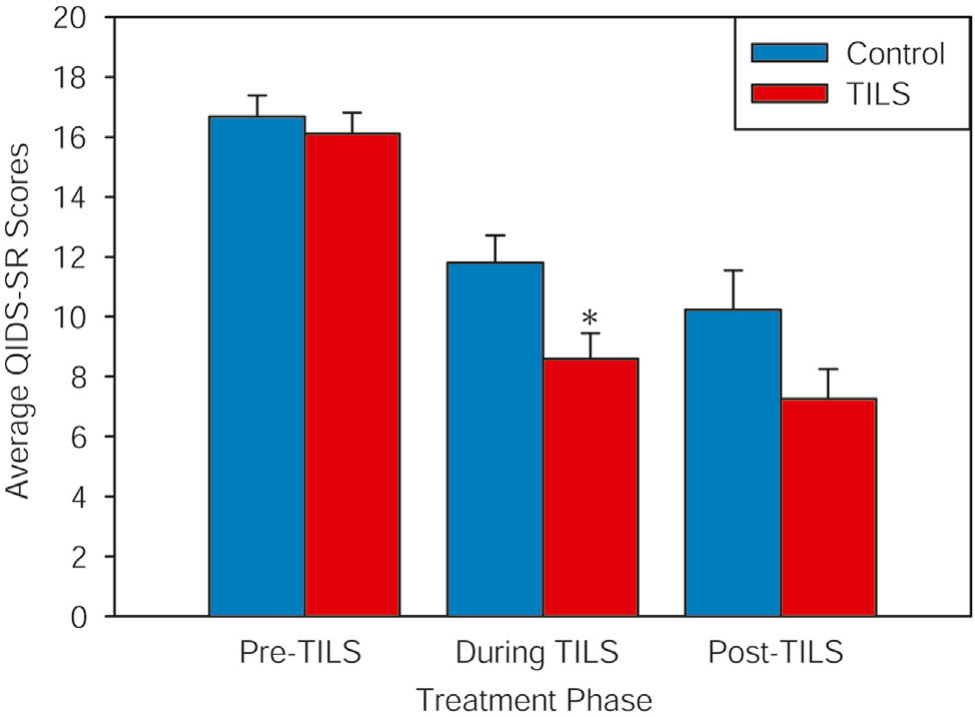
Average (mean ± SE) depression symptom scores (Quick Inventory of Depressive Symptomatology–Self-Report [QIDS-SR]) in the 3 treatment phases: pre-transcranial infrared laser stimulation (TILS) (week 0), during TILS (weeks 3–6), and post-TILS (weeks 7–12). TILS significantly reduced depression symptoms in the during-TILS phase (∗uncorrected *p* = .016, Bonferroni-corrected *p* < .05).

Average QIDS-SR scores for the 2 groups were also analyzed separately during the 3 phases of the experiment using the mean imputation approach. Figure 2 shows that during the pre-TILS phase, there were no significant group differences in QIDS-SR scores (2-tailed *t* test, *t*_38_ = 0.75, *p* = .46), supporting that there were no preexisting differences in depression scores between groups before the start of the experiment. In the during-TILS phase, there was a significant reduction in QIDS-SR scores in the TILS-treated group (2-tailed *t* test, *t*_38_ = 2.53, *p* = .016, Cohen’s *d* = 0.80). In the post-TILS phase, there was a nonsignificant trend for a mean reduction in QIDS-SR scores in the group that had received TILS compared with the sham-treated group (2-tailed *t* test, *t*_29_ = 1.82, *p* = .079, Cohen’s *d* = 0.65).

### Weekly Effects During and After TILS Treatment

**Figure 3:**
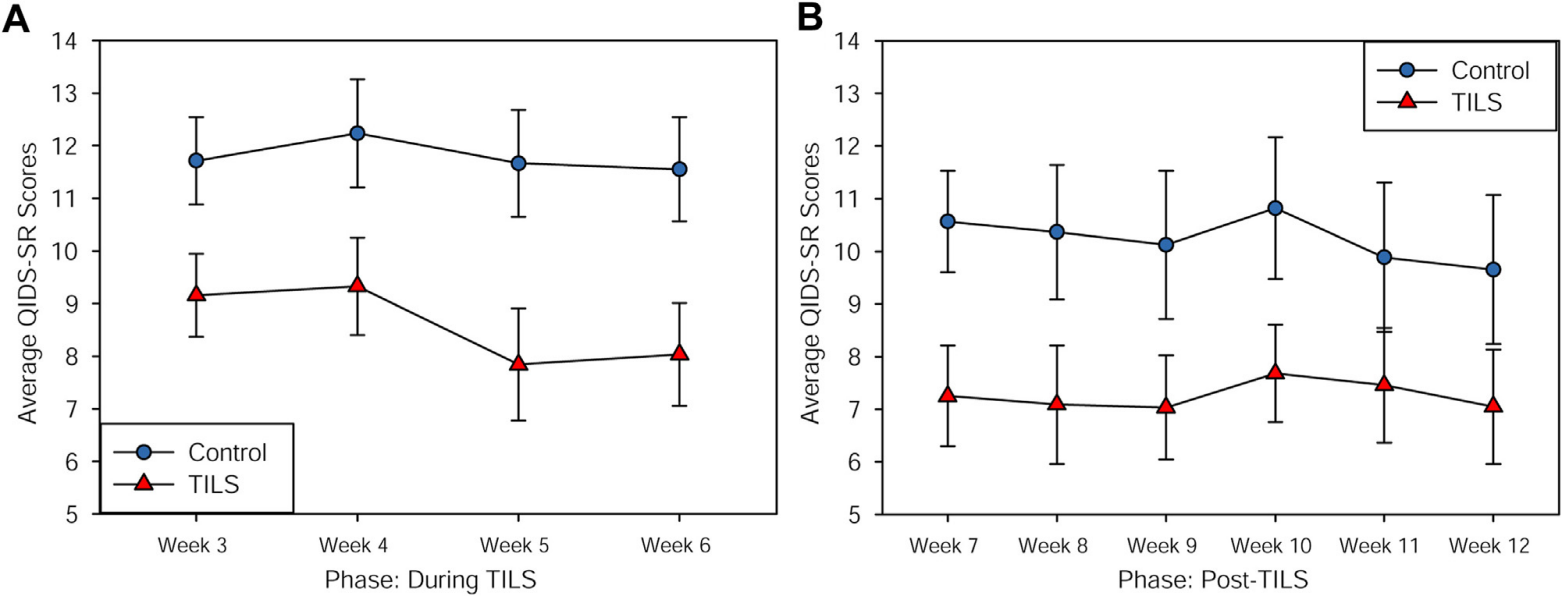
Average (mean ± SE) depression symptom scores (Quick Inventory of Depressive Symptomatology–Self-Report [QIDS-SR]) **(A)** during the transcranial infrared laser stimulation (TILS)/sham treatment phase (weeks 3–6) and **(B)** after the TILS/sham treatment phase (weeks 7–12).

We further analyzed the weekly group differences during and after TILS or sham treatment using repeated-measures ANOVA (2 factors: weeks and group). During TILS, there was a significant effect of group, with the TILS group showing lower QIDS-SR scores than the sham control group (*F*_1,38_ = 6.39, *p* = .016, η_p_^2^ = 0.144). There was not a significant main effect of weeks (*p* = .112) or a significant interaction of weeks × group (*p* = .58), because the rate of change in both groups during TILS and sham treatments resulted in 2 similar parallel lines (Figure 3A).

In the post-TILS phase, there was a nonsignificant trend for a mean reduction in QIDS-SR scores in the group that had received TILS compared with the sham-treated group (*F*_1,29_ = 3.31, *p* = .079, η_p_^2^ = 0.102). There was not a significant main effect of weeks (*p* = .48) or a significant interaction of weeks × group (*p* = .89), because the rate of change in both groups after TILS and sham treatments resulted in 2 similar parallel lines (Figure 3B).

## Discussion

The results showed that the effectiveness of Deprexis in decreasing depressive symptoms was augmented by the laser treatment. While Deprexis alone significantly reduced depression scores in the placebo control group, this beneficial effect was augmented with the addition of TILS as an adjunct therapy. Both the TILS and control groups received Deprexis, which resulted in a gradual decrease in depression symptoms in both groups (approximately parallel lines), and therefore a significant interaction of weeks × group was not found.

To handle missing values, we used a multiple imputation statistical approach by creating multiple datasets with different imputed values and then analyzed each dataset separately. The presentation of the results was combined to account for the uncertainty of the imputations. We performed data analyses under different statistical assumptions about the missing data. All the methods used revealed significant main effects of Deprexis and TILS treatments, with nearly identical probability values and effect sizes. Therefore, we found that our results were robust to different handling methods.

These results are consistent with our previous findings that TILS can have a beneficial effect on depression symptoms when combined with a cognitive intervention such as ABM (14). This previous work found that TILS to the right PFC led to a greater reduction of depression symptoms after ABM than stimulation to the left PFC or sham. TILS to the right PFC also had beneficial brain metabolic and cognitive effects in our other studies using euthymic participants (9, 10, 11, 12, 13). However, in Disner *et al.* (14), cognitive response to ABM moderated the impact of neuroenhancement. It is for this reason that we limited the participant pool to individuals who showed an initial 10% improvement in depression symptoms from Deprexis. We hoped to capture individuals who showed cognitive responsiveness to Deprexis because they may be more likely to show a synergistic effect between Deprexis and TILS in reducing depression symptoms.

TILS treatment may also be useful as a monotherapy in treating anxiety disorders. In a study of the effects of exposure therapy on the treatment of pathological fears (19), we found that TILS administered to the dorsolateral PFC was successful in reducing context-specific fear. However, TILS administered to the ventromedial PFC did not significantly reduce fear, either alone or in conjunction with exposure therapy. While the stimulation parameters (apparatus, irradiance, energy density) (21) were identical to those used in the current study, the treatment was delivered bilaterally instead of only on the right side. Thus, in Zaizar *et al.* (19), the PFC only received half of the energy dose used in the current study. Unilateral stimulation of the dorsolateral PFC with near-infrared light at 810 nm also resulted in significantly decreased depression and anxiety symptoms in participants with MDD as well as increased frontal cerebral blood flow (22).

The PFC has been implicated in emotional regulation (23), and in the right hemisphere, the PFC is a major hub for outgoing connections to other brain regions (24). Frontal regions also showed decreased energy metabolism in an animal model of depression (25), which is consistent with findings in humans (26), with recovery from depression being associated with metabolic increases in the PFC (27). Reduced top-down control of subcortical regions that mediate emotional processes may play a role in depression (28), so augmenting PFC function with TILS may restore an inhibitory influence that is otherwise reduced in depression. Increased activity in the right frontal pole is associated with improvements in reappraisal after CBT (29). Therefore, augmentation of this region’s metabolic activity with TILS may help participants undergoing CBT to more effectively use newly learned adaptive coping strategies.

The mechanisms of transcranial photobiomodulation relevant to depression have been reviewed recently by Vieira *et al.* (16,30). Briefly, they include 1) activation of mitochondrial complexes and enhanced ATP (adenosine triphosphate) production through increased activity in the PFC, 2) normalization of blood sugar and cortisol levels in the brain cortex, 3) reduction in cortisol levels, 4) increased serotonin levels, 5) modulation of nitric oxide in the PFC, 6) modulation of the glutamatergic system, 7) decrease in monoamine oxidase levels, 8) reduction in monoamine depletion, 9) decrease in lipid peroxidation, and 10) increased expression of BDNF (brain-derived neurotrophic factor) and TrkB (tropomyosin receptor kinase B).

Most mechanisms of transcranial photobiomodulation appear to be primarily based on near-infrared light that modulates brain function through mitochondrial bioenergetics, a mechanism distinct from other brain stimulation methods such as electrical or magnetic stimulation. Specifically, TILS has been demonstrated to modulate neuronal function, cognitive abilities, and emotional states by using red to near-infrared light to activate cytochrome c oxidase and oxygenated hemoglobin in human brains, which enhances mitochondrial energy production (31). This process increases ATP production, nitric oxide, and blood oxygenation, supporting emotional and cognitive functions based in the PFC (8). The stimulation of cytochrome oxidase by TILS not only provides immediate energy but also leads to a long-term increase in cytochrome c oxidase levels, which boosts brain metabolism and improves neuroplasticity (32). These metabolic and hemodynamic mechanisms may be beneficial for alleviating symptoms of depression because patients with depression are characterized by a hypometabolic PFC.

In addition, antioxidant and anti-inflammatory photobiomodulation mechanisms (33) may play a role in diminishing some of the pathophysiology found in depression (30). For example, biomarkers of oxidative stress, inflammation, and tryptophan catabolites are elevated in depression. Mitochondrial oxidative processes generate reactive oxygen species (ROS) that, when excessive, can damage cell components such as DNA, proteins, and fatty acids, contributing to depression through mechanisms such as inflammation and neurodegeneration. ROS trigger the release of proinflammatory cytokines that can disrupt the blood-brain barrier, impair hippocampal neurogenesis, and affect brain connectivity. Brain regions with high metabolic rates are particularly vulnerable to oxidative damage. Studies have also shown that individuals with depression exhibit lower antioxidant levels and higher markers of oxidative stress, suggesting heightened oxidative processes in depression. Photobiomodulation has been shown to reduce oxidative stress, although the exact mechanisms are not fully understood (30). TILS can increase ATP synthesis, mitochondrial membrane potential, and calcium levels in healthy neurons, with some studies suggesting that it induces short-term ROS production in mitochondria. This burst of ROS may trigger the release of antioxidants such as superoxide dismutase and catalase, which could be beneficial in conditions in which antioxidant defenses are impaired, such as depression.

A study limitation was that participants could access the Deprexis website as often as they liked. The Deprexis program is self-guided, so participants determine how often they access the material. However, this information and concomitant treatments could not be monitored as part of the study, because the institutional review board restricted the researchers’ ability to collect online information about participants due to privacy and confidentiality concerns related to using online resources.

### Conclusions

The results of this study indicate that TILS may serve as a useful adjunct therapy to CBT for the treatment of MDD. The combination of TILS with other neuromodulation approaches (34) may also help improve MDD treatment. Our previous findings of cognitive enhancement and increased brain metabolic activity in euthymic participants may also be found in the MDD patient population, as we have seen in patients with bipolar depression (35). Further research that pairs neuroenhancement methods with cognitive interventions may reveal the potential to improve treatment outcomes in depression and other psychiatric disorders.

## Supplementary Material

Key Resources Table
